# An Extended Damage Plasticity Model for Shotcrete: Formulation and Comparison with Other Shotcrete Models

**DOI:** 10.3390/ma10010082

**Published:** 2017-01-21

**Authors:** Matthias Neuner, Peter Gamnitzer, Günter Hofstetter

**Affiliations:** Unit for Strength of Materials and Structural Analysis, Institute of Basic Sciences in Engineering Science, Innsbruck University, Technikerstr. 13, A-6020 Innsbruck, Austria; Peter.Gamnitzer@uibk.ac.at (P.G.); Guenter.Hofstetter@uibk.ac.at (G.H.)

**Keywords:** shotcrete, constitutive model, experimental data, tunnel advance, numerical simulation

## Abstract

The aims of the present paper are (i) to briefly review single-field and multi-field shotcrete models proposed in the literature; (ii) to propose the extension of a damage-plasticity model for concrete to shotcrete; and (iii) to evaluate the capabilities of the proposed extended damage-plasticity model for shotcrete by comparing the predicted response with experimental data for shotcrete and with the response predicted by shotcrete models, available in the literature. The results of the evaluation will be used for recommendations concerning the application and further improvements of the investigated shotcrete models and they will serve as a basis for the design of a new lab test program, complementing the existing ones.

## 1. Introduction

The use of shotcrete for supporting the surrounding rock or soil of a tunnel is an essential part of the New Austrian Tunneling method (NATM). Hence, for numerical simulations of tunnel advance based on the NATM, in addition to a constitutive model for the rock mass, a constitutive model for shotcrete is required for representing the mechanical behavior of the shotcrete shell. Shotcrete is characterized by continuously developing material properties during hydration, nonlinear stress–strain relations, time-dependent material behavior due to creep and shrinkage and fracture. In contrast to concrete, shotcrete applied for securing excavations in tunneling is already loaded at very early ages. Compared to the large body of literature on experimental investigations and constitutive modeling of concrete, only a relatively small number of experiments, focusing on the characterization of the complex material behavior of shotcrete, and only a few constitutive models for shotcrete have been reported in the literature up to now.

An early attempt to account for the time-dependent shotcrete behavior in numerical simulations of tunnel advance was proposed in [1]. In this practical approach, a reduced Young’s modulus for shotcrete is used to consider effects such as evolving stiffness and strength during hydration, creep, shrinkage and even effects due to time-dependent loading caused by consecutive excavation steps. By combining these effects in a hypothetical modulus of elasticity (HME), approximate axisymmetric solutions for tunneling can be obtained. Problematic is the lack of calibration parameters for the model in the literature, and thus, the magnitude of the HME is commonly based only on experience.

Constitutive models for shotcrete, formulated within the framework of continuum mechanics, can be broadly subdivided into single-field and multi-field models. The former only focus on the mechanical behavior by providing time-dependent stress–strain relations, whereas the latter account for interactions between the mechanical behavior and chemical, thermal and hygral processes, related to hydration and influenced by the ambient conditions.

Representatives of the single-field shotcrete models are the shotcrete models, proposed by Meschke [2], by Schütz et al. [3] and, partially based on the latter, the recently proposed shotcrete model by Schädlich and Schweiger [4]. The single-field shotcrete models have in common the description of the evolution of material stiffness and strength in terms of the shotcrete age, the application of a yield surface for delimiting the domain of elastic material behavior and consideration of hardening and/or softening material behavior, the latter being regularized within the framework of the Finite Element Method (FEM) by means of the specific fracture energy and a characteristic element length. Main differences of the models proposed in [2,4], are related to the applied type of the flow rule and to the employed theory for modeling creep.

In the single-field shotcrete models, several phenomena, such as the evolution of mechanical properties, shrinkage and creep are described separately by empirical relations, ignoring interactions among them. Distinctions between autogenous shrinkage and drying shrinkage and between basic creep and drying creep are only made indirectly by adjusting the ultimate shrinkage strain or the creep coefficient. Furthermore, hydration and aging of shotcrete do not only depend on the shotcrete age but also on the water content of shotcrete and on temperature. Temperature, in turn, is increased by hydration whereas the water content is decreased. Both temperature and water content also depend on the ambient conditions. Autogenous shrinkage depends on hydration. Drying shrinkage is caused by the increase of capillary stress, acting in the unsaturated pores of the porous material concrete, due to the decrease of the degree of water saturation in the pores of the porous material concrete. Creep depends on hydration and moisture content, in addition to the acting sustained stress. The increase of capillary stress due to the decrease in moisture content in the pores is related to the Pickett effect [5] observed in drying creep. Hence, it follows that contrary to the simplifying assumptions inherent in the single-field models, these phenomena are actually coupled and because of the interactions and the dependency on the ambient conditions, the material properties are not uniformly distributed in a shotcrete shell.

For the above reasons, an alternative to single-field shotcrete models are multi-field models. In the latter, concrete is described as a porous material, consisting of several interacting phases, namely the solid phase with an embedded pore structure, containing one or several fluid phases (water phase, dry air phase and vapor phase). The first multi-field model for shotcrete was proposed by Hellmich et al. [6]. Considering the dependency of shotcrete properties on the degree of hydration, i.e., on the time- and temperature-dependent chemical reaction between cement and water, it is based on thermo–chemo-plasticity theory along the lines of the thermo–chemo-mechanical framework, developed by Ulm and Coussy [7]. This model was further developed in [8] to consider early age cracking of shotcrete. An even broader framework for concrete was proposed by Gawin et al. [9,10] by including hygral effects, resulting in a hygro–thermo–chemo-mechanical concrete model. The latter was modified by several authors, e.g., recently by Sciume et al. [11] for modeling repair work.

Similar to the available literature on shotcrete models, also the number of experimental studies on the early age behavior of shotcrete is limited up to the present day. Hence, it is not surprising that most of the mentioned shotcrete models were calibrated mainly on the basis of the same sets of results of laboratory tests and, due to the limited experimental data for shotcrete, frequently experimental results for normal concrete were used for calibration. Results of experimental programs, specifically devoted to shotcrete, are documented in the following publications:

- Sezaki et al. [12] published test results on the evolution of Young’s modulus and uniaxial compressive strength up to the age of 28 days and stress–strain relations from short-term uniaxial and triaxial compression tests on specimens of different age.
- Aldrian [13] and Golser et al. [14] presented experimental results on the evolution of the uniaxial compressive strength up to the age of 28 days, results of uniaxial compression tests and of the evolution of the total strain in creep and shrinkage tests.
- Huber [15] investigated the evolution of temperature due to hydration, of the Young’s modulus and of the uniaxial compressive strength up to the age of 7 days as well as the evolution of the total strain in shrinkage and creep tests.
- Fischnaller [16] presented test results on the evolution of Young’s modulus and uniaxial compressive strength and the results of relaxation and shrinkage tests up to the age of 7 days.
- Müller [17] published test results on the evolution of stiffness and strength, results of short-term uniaxial compression tests and the evolution of the total strain in shrinkage and creep tests.

In the mentioned laboratory tests on the shrinkage behavior of shotcrete, conducted on sealed and unsealed specimens, only the evolution of the total strain is reported. Thus, in the case of sealed specimens, the measured strain consists of the autogenous shrinkage strain and the thermally induced strain due to the hydration heat for very early shotcrete ages. In the case of unsealed specimens, furthermore the drying shrinkage strain is included in the measured total strain. Similarly, the reported measured total strain of creep tests includes a combination of strain components induced by different phenomena. In the case of creep tests on sealed specimens, they consist of the basic creep strain in addition to the strain components measured in the shrinkage tests on sealed specimens. In the case of creep tests on unsealed specimens, they include the basic creep strain and drying creep strain in addition to the strain components measured in the shrinkage tests on unsealed specimens.

The paper is organized as follows. In the next section, the damage-plasticity model for concrete, proposed by Grassl and Jirásek [18], is extended to meet the specific requirements of a constitutive model for shotcrete concerning the time-dependent evolution of material properties and of the strain due to creep and shrinkage. In Section 3, the performance of the proposed extended damage plasticity model for shotcrete is evaluated by comparing the predicted response with experimental data and with the response of the shotcrete models proposed by Meschke [2] and by Schädlich et al. [4] and with the response of the multi-field concrete model, proposed by Gawin et al. [9,10]. In the interests of brevity, equations for the latter models will not be provided as they are available in the respective papers for the interested reader. The comparison of experimental and numerical results will be used for recommendations concerning the application and further improvement of the investigated shotcrete models and it will serve as a basis for the design of a new lab test program, complementing the existing ones.

## 2. An Extended Damage Plasticity Model for Shotcrete

The damage plasticity model serves as the starting point for the formulation of a constitutive model for shotcrete, proposed by Grassl and Jirásek [18] for describing the time-independent material behavior of concrete. The model is here denoted as the Concrete Damage Plastic (CDP) model. In the latter, the nonlinear mechanical behavior of concrete is modeled by means of a combination of plasticity theory and the theory of damage mechanics. The smooth yield surface is expanding during hardening until at peak stress it attains the triaxial strength envelope for concrete, proposed by Menétrey and Willam [19]. The plastic strain rate is determined by means of a non-associated flow rule. Beyond peak strength, stiffness degradation and softening material behavior are described by means of an isotropic damage model.

The CDP model is extended by considering the aging of shotcrete and creep by means of the solidification theory [20] and shrinkage on the basis of the Bažant–Panula model [21]. To improve the representation of the evolution of the material properties of shotcrete, especially up to the age of 24 h, several modifications are proposed. The extended model is denoted as Shotcrete Damage Plasticity (SCDP) model.

The solidification theory is incorporated into the damage-plasticity framework by modeling creep in the effective stress space. The nonlinear stress–strain relation is given in total form as

(1) $$\sigma =\left(1-\omega \right)C:\left(\varepsilon -\varepsilon^{p}-\varepsilon^{\mathrm{ve}}-\varepsilon^{f}-\varepsilon^{\mathrm{shr}}\right).$$

The total strain *ε* is decomposed into the elastic strain $\varepsilon^{\mathrm{el}}=\varepsilon -\varepsilon^{p}-\varepsilon^{\mathrm{ve}}-\varepsilon^{f}-\varepsilon^{\mathrm{shr}}$, the plastic strain $\varepsilon^{p}$, the (due to aging only partially recoverable) viscoelastic strain $\varepsilon^{\mathrm{ve}}$, the flow strain $\varepsilon^{f}$ and the shrinkage strain $\varepsilon^{\mathrm{shr}}$. The nominal stress tensor *σ* is related to the effective stress tensor $\bar{\sigma }$ by the isotropic scalar variable *ω* as

(2) $$\sigma =\left(1-\omega \right)\bar{\sigma }.$$

To account for the evolution of material properties of shotcrete, especially during the hydration, several extensions and improvements are presented. They comprise the modification of the volume function v(t) of the solidification theory and of the ductility formulation of the CDP model and the proposal of a law for the evolution of the material strength.

### 2.1. Damage Plasticity Framework

The plastic response, governed by the yield function for delimiting the elastic domain, the plastic potential function for controlling the evolution of the plastic strain and the hardening law are adopted from the CDP model [18]. In contrast to the latter, the uniaxial compressive strength $f_{\mathrm{cu}}\left(t\right)$, the uniaxial yield stress $f_{\mathrm{cy}}\left(t\right)$, the biaxial compressive strength $f_{\mathrm{cb}}\left(t\right)$, the uniaxial tensile strength $f_{\mathrm{tu}}\left(t\right)$ and the plastic strain $\varepsilon_{\mathrm{cpu}}^{p}\left(t\right)$ at uniaxial compressive peak stress are assumed to be dependent on time *t*. Hence, the time-independent yield function $f_{p}$ and the plastic potential $g_{p}$ of the CDP model are replaced by

(3) $$\begin{array}{c} \begin{array}{cc} f_{p}\left({\bar{\sigma }}_{m},\bar{\rho },\theta ,q_{h}\left(\alpha_{p}\right),t\right)= & \left(1-q_{h}\left(\alpha_{p}\right)\right)\left(\frac{\bar{\rho }}{\sqrt{6}f_{\mathrm{cu}}\left(t\right)}+\frac{{\bar{\sigma }}_{m}}{f_{\mathrm{cu}}\left(t\right)}\right)+\sqrt{\frac{3}{2}}{\left(\frac{\bar{\rho }}{f_{\mathrm{cu}}\left(t\right)}\right)}^{2}\\ & +m_{0}q_{h}^{2}\left(\alpha_{p}\right)\left(\frac{\bar{\rho }}{\sqrt{6}f_{\mathrm{cu}}\left(t\right)}r\left(\theta \right)+\frac{{\bar{\sigma }}_{m}}{f_{\mathrm{cu}}\left(t\right)}\right)-q_{h}^{2}\left(\alpha_{p}\right), \end{array} \end{array}$$

(4) $$\begin{array}{c} \begin{array}{cc} g_{p}\left({\bar{\sigma }}_{m},\bar{\rho },\alpha_{p},q_{h}\left(\alpha_{p}\right),t\right)= & \left(1-q_{h}\left(\alpha_{p}\right)\right)\left(\frac{\bar{\rho }}{\sqrt{6}f_{\mathrm{cu}}\left(t\right)}+\frac{{\bar{\sigma }}_{m}}{f_{\mathrm{cu}}\left(t\right)}\right)\\ & +\sqrt{\frac{3}{2}}{\left(\frac{\bar{\rho }}{f_{\mathrm{cu}}\left(t\right)}\right)}^{2}q_{h}^{2}\left(\alpha_{p}\right)\left(\frac{m_{0}\bar{\rho }}{\sqrt{6}f_{\mathrm{cu}}\left(t\right)}+\frac{m_{g}\left({\bar{\sigma }}_{m}\right)}{f_{\mathrm{cu}}\left(t\right)}\right). \end{array} \end{array}$$

They are formulated in terms of three invariants of the effective stress tensor, i.e., the effective mean stress ${\bar{\sigma }}_{m}$, the effective deviatoric radius $\bar{\rho }$ and the Lode angle *θ*, and the stress-like internal variable $q_{h}$, the latter in terms of the strain-like internal variable $\alpha_{p}$. r(θ) describes the shape of the yield surface in deviatoric planes, and $m_{0}$ and $m_{g}$ are model parameters, dependent on the material parameters $f_{\mathrm{cu}}\left(t\right)$, $f_{\mathrm{cy}}\left(t\right)$, $f_{\mathrm{cb}}\left(t\right)$ and $f_{\mathrm{tu}}\left(t\right)$. The rate of the plastic strain is given as

(5) $${\dot{\varepsilon }}^{p}=\dot{\lambda }\frac{\partial g_{p}\left({\bar{\sigma }}_{m},\bar{\rho },q_{h}\left(\alpha_{p}\right),t\right)}{\partial \bar{\sigma }}$$

and the modified evolution law for $\alpha_{p}$ is adopted from [22]:

(6) $${\dot{\alpha }}_{p}=∥{\dot{\varepsilon }}^{p}\left(t\right)∥\frac{1}{x_{h}\left({\bar{\sigma }}_{m},t\right)}\left(1+3\frac{{\bar{\rho }}^{2}}{{\bar{\rho }}^{2}+\vartheta_{h}}\cos^{2}\left(1.5\theta \right)\right),$$

with $\vartheta_{h}$ as a small disturbance parameter to avoid division by zero in the case of hydrostatic stress. The ductility function $x_{h}\left({\bar{\sigma }}_{m}\right)$ controls the magnitude of ${\dot{\alpha }}_{p}$ dependent on the acting hydrostatic stress:

(7) $$\begin{array}{c} x_{h}\left({\bar{\sigma }}_{m}\right)=\{\begin{array}{cc} A_{h}-\left(A_{h}-B_{h}\right)\exp\left(\frac{-R_{h}\left({\bar{\sigma }}_{m}\right)}{C_{h}}\right) & \mathrm{if}\;R_{h}\left({\bar{\sigma }}_{m}\right)\geq 0,\\ \left(B_{h}-D_{h}\right)\exp\left(\frac{R_{h}\left({\bar{\sigma }}_{m}\right)\left(A_{h}-B_{h}\right)}{\left(B_{h}-D_{h}\right)C_{h}}\right)+D_{h} & \mathrm{otherwise}, \end{array}\\ R_{h}\left({\bar{\sigma }}_{m}\right)=-\frac{{\bar{\sigma }}_{m}}{f_{\mathrm{cu}}}-\frac{1}{3}, \end{array}$$

in which $A_{h}$, $B_{h}$, $C_{h}$ and $D_{h}$ are model parameters, which are calibrated from uniaxial tensile and uniaxial and triaxial compressive tests. Assuming fixed values of $A_{h}$, $C_{h}$ and $D_{h}$, the material ductility in the pre-peak regime of the stress–strain relation is governed by the model parameter $B_{h}$. Although the actual relation between $\varepsilon_{\mathrm{cpu}}^{p}$—the plastic strain at peak stress in uniaxial compression—and $B_{h}$ is nonlinear, an approximative linear relation is proposed by Grassl and Jirásek. However, this linear relation is valid only for a limited range of $\varepsilon_{\mathrm{cpu}}^{p}($, from 0 to approximately -0.002. To extend the valid range of the approximative law, an extension is proposed as

(8) $$B_{h}\left(\varepsilon_{\mathrm{cpu}}^{p}\right)=\left\{\begin{array}{cc} -2.29\;\varepsilon_{\mathrm{cpu}}^{p}+0.00046 & \mathrm{if}\;\varepsilon_{\mathrm{cpu}}^{p}>-0.002,\;\\ -1.55\;(\varepsilon_{\mathrm{cpu}}^{p}+0.002)+0.00504 & \mathrm{if}\;\varepsilon_{\mathrm{cpu}}^{p}\leq -0.02. \end{array}\right.$$

The evolution law for $\varepsilon_{\mathrm{cpu}}^{p}\left(t\right)$ is adopted from the model by Schädlich and Schweiger [4], as experimental results for a more refined law are not available: It is based on linear interpolation of $\varepsilon_{\mathrm{cpu}}^{p}$ between the values at the age of 1 h, 8 h and 24 h, denoted as $\varepsilon_{\mathrm{cpu}}^{p(1)}$, $\varepsilon_{\mathrm{cpu}}^{p(8)}$ and $\varepsilon_{\mathrm{cpu}}^{p(24)}$, respectively. For t<1 h and t>24 h, $\varepsilon_{\mathrm{cpu}}^{p}$ is assumed to be constant.

The softening behavior is adopted from the CDP model [18], relating the isotropic damage variable *ω* to the internal strain-like softening variable $\alpha_{d}$ by the exponential softening law

(9) $$\omega =1-\exp\left(-\frac{\alpha_{d}}{\varepsilon_{f}}\right).$$

$\varepsilon_{f}$ is the softening modulus, controlling the slope of the softening curve. The evolution of the strain-like internal variable $\alpha_{d}$ is related to the rate of the volumetric plastic strain ${\dot{\varepsilon }}_{V}^{p}$ by

(10) $$\begin{array}{cc} {\dot{\alpha }}_{d} & =\left\{\begin{array}{cc} 0 & \mathrm{if}\;\alpha_{p}<1,\;\\ {\dot{\varepsilon }}_{V}^{p}/x_{s}\left({\dot{\varepsilon }}^{p}\right) & \mathrm{otherwise}. \end{array}\right. \end{array}$$

Function $x_{s}\left({\dot{\varepsilon }}^{p}\right)$ represents a ductility measure:

(11) $$x_{s}\left({\dot{\varepsilon }}^{p}\right)=\left\{\begin{array}{cc} 1+A_{s}\;R_{s}^{2}\left({\dot{\varepsilon }}^{p}\right) & \mathrm{if}\;R_{s}\left({\dot{\varepsilon }}^{p}\right)<1,\\ 1-3\;A_{s}+4\;A_{s}\sqrt{R_{s}\left({\dot{\varepsilon }}^{p}\right)} & \mathrm{otherwise}. \end{array}\right.$$

$A_{s}$ is a model parameter to be calibrated from uniaxial tensile tests and $R_{s}\left({\dot{\varepsilon }}^{p}\right)$ denotes the ratio of the *negative* volumetric plastic strain rate to the total volumetric plastic strain rate:

(12) $$R_{s}\left({\dot{\varepsilon }}^{p}\right)=\frac{{\dot{\varepsilon }}_{V}^{p⊝}}{{\dot{\varepsilon }}_{V}^{p}}\;\mathrm{with}\;{\dot{\varepsilon }}_{V}^{p⊝}=\sum_{i=I}^{\mathrm{III}}\langle -{\dot{\varepsilon }}_{\left(i\right)}^{p}\rangle ,$$

the latter computed from the principal values of the plastic strain rate tensor.

### 2.2. Evolution of Material Strength

The evolution of $f_{\mathrm{cu}}\left(t\right)$ is described by a modified version of the law originally proposed in [23] for the evolution of the Young’s modulus:

(13) $$f_{\mathrm{cu}}\left(t\right)=f_{\mathrm{cu}}^{\left(28\right)}\;\beta_{f}\left(t\right),\;\beta_{f}\left(t\right)=\{\begin{array}{cc} \beta_{f}^{I}=r_{f}+c_{f}t+d_{f}t^{2} & \mathrm{if}\;t\leq t_{f},\\ \beta_{f}^{\mathrm{II}}={\left(a_{f}+\frac{b_{f}}{t-\Delta t_{f}}\right)}^{-0.5} & \mathrm{if}\;t_{f}<t\leq 28\;d,\\ \beta_{f}^{\mathrm{III}}=1 & \mathrm{otherwise}. \end{array}$$

Supplementing the original formulation in [23], a residual parameter $r_{f}$ ensures a non-zero compressive strength already at zero age. A default value is proposed as $r_{f}=10^{-2}$, resulting in a uniaxial compressive strength of 1 % of $f_{\mathrm{cu}}^{\left(28\right)}$ at t=0. The model parameters are given as

(14) $$\begin{array}{cccc} a_{f} & =\frac{1-\frac{28-\Delta t_{f}}{1-\Delta t_{f}}{\left(f_{\mathrm{cu}}^{\left(1\right)}/f_{\mathrm{cu}}^{\left(28\right)}\right)}^{2}}{(1-\frac{28-\Delta t_{f}}{1-\Delta t_{f}}){\left(f_{\mathrm{cu}}^{\left(1\right)}/f_{\mathrm{cu}}^{\left(28\right)}\right)}^{2}},\; & b_{f} & =\left(28-\Delta t_{f}\right)\left(1-a_{f}\right),\\ c_{f} & =\frac{d\beta_{f}^{\mathrm{II}}}{dt}{|}_{t=t_{f}}-\frac{2\beta_{f}^{\mathrm{II}}}{t_{f}},\; & d_{f} & =\left(\frac{d\beta_{f}^{\mathrm{II}}}{dt}{|}_{t=t_{f}}\;t_{f}-\left(\beta_{f}^{\mathrm{II}}-r_{f}\right)\right)/t_{f}^{2}. \end{array}$$

$f_{\mathrm{cu}}^{\left(1\right)}$ and $f_{\mathrm{cu}}^{\left(28\right)}$ denote the experimentally determined values for $f_{\mathrm{cu}}\left(t\right)$ at the age of 1 day and 28 days, respectively. Parameters $t_{f}$ and $\Delta t_{f}$ (given in days) control the delayed start of the evolution of compressive strength. Default values are proposed as $t_{f}=0.25\;d$ and $\Delta t_{f}=0.18\;d$. Employing these values, monotonic growth of $f_{\mathrm{cu}}\left(t\right)$ is ensured only for ratios $f_{\mathrm{cu}}^{\left(1\right)}/f_{\mathrm{cu}}^{\left(28\right)}\geq 0.16$, which are usually met in practical applications.

Regarding $f_{\mathrm{cy}}\left(t\right)$, $f_{\mathrm{cb}}\left(t\right)$ and $f_{\mathrm{tu}}\left(t\right)$, it is assumed that ratios $f_{\mathrm{cy}}\left(t\right)/f_{\mathrm{cu}}\left(t\right)$, $f_{\mathrm{cb}}\left(t\right)/f_{\mathrm{cu}}\left(t\right)$ and $f_{\mathrm{tu}}\left(t\right)/f_{\mathrm{cu}}\left(t\right)$ remain constant throughout the hydration process, and thus, they are equal to the respective ratios at the age of 28 days.

### 2.3. Aging Material Behavior and Creep Strain

The evolution laws for the elastic strain, the viscoelastic strain and the flow strain are adopted from the solidification theory for concrete aging and creep [20]. Since they are incorporated into the damage plasticity model, they are formulated in the effective stress space:

(15a) $$\begin{array}{cc} {\dot{\varepsilon }}^{\mathrm{el}}\left(t\right) & =q_{1}C_{\nu }^{-1}:\dot{\bar{\sigma }}\left(t\right), \end{array}$$

(15b) $$\begin{array}{cc} {\dot{\varepsilon }}^{\mathrm{ve}}\left(t\right) & =\frac{F(\bar{\sigma }\left(t\right))}{v(t)}\;\int_{0}^{t}\dot{\Phi }\left(t-t^{\prime }\right)C_{\nu }^{-1}:d\bar{\sigma }\left(t^{\prime }\right), \end{array}$$

(15c) $$\begin{array}{cc} {\dot{\varepsilon }}^{f}\left(t\right) & =\frac{q_{4}F\left(\bar{\sigma }\left(t\right)\right)}{t}C_{\nu }^{-1}:\bar{\sigma }\left(t\right). \end{array}$$

$C_{\nu }$ denotes the elastic unit stiffness tensor [24]. The volume function v(t), describing the evolution of the load bearing volume fraction of the hydrated material, and function $\Phi (t-t^{\prime })$ are given as

(16) $$\begin{array}{cc} v(t) & ={\left({\left(\frac{1}{t}\right)}^{0.5}+\frac{q_{3}}{q_{2}}\right)}^{-1}, \end{array}$$

(17) $$\begin{array}{cc} \Phi (t-t^{\prime }) & =q_{2}\;\ln\left(1+{\left(t-t^{\prime }\right)}^{0.1}\right). \end{array}$$

$q_{1}$, $q_{2}$, $q_{3}$ and $q_{4}$ in (15)–(17) are the compliance parameters.

Function $F(\bar{\sigma }\left(t\right))$ in (15) controls the nonlinear dependency of the creep strain rate on the acting effective stress. Adapting the proposed expression for F(σ(t)) for uniaxial compressive states in [20] to effective stresses yields

(18) $$\begin{array}{cc} F(\bar{\sigma }\left(t\right)) & =1+s{\left(t\right)}^{2}, \end{array}$$

with $s\left(t\right)=\bar{\sigma }\left(t\right)/f_{\mathrm{cu}}\left(t\right)$ denoting the ratio of the acting effective uniaxial compressive stress $\bar{\sigma }\left(t\right)$ over $f_{\mathrm{cu}}\left(t\right)$. Equation (18) is sufficient for the numerical simulation of the uniaxial creep tests, presented in Section 3. However, the extension to multi-axial stress states is pending. The compliance parameters $q_{1}$ to $q_{4}$ may be computed according to the estimation procedures presented in [25,26], or alternatively they are calibrated on the basis of experimental data. However, even if $q_{2}$ and $q_{3}$ are identified from experimental results, the evolution of the Young’s modulus of shotcrete at very early ages is not well represented by v(t). Especially up to the age of approximately 3 h, the Young’s modulus is overestimated, whereas beyond the age of 3 h the rapid evolution of Young’s modulus is heavily underestimated. Indeed, this fact is not surprising as neither the solidification theory nor the respective parameter estimation procedure are intended to represent the material behavior at such early material ages. Furthermore, usually shotcrete exhibits an increased hydration speed compared to normal concrete due to an added accelerator. Hence, v(t) is modified based on a time transformation, by analogy to the methods used in [27] to account for temperature effects on the evolution of the creep strain. To this end, v(t) is replaced by v(τ(t)) with τ(t) as a time transformation function.

The proposed modification is characterized by (i) an improved representation of the initially low stiffness up to approximately 3 h, which is overestimated by the original volume function; (ii) an increased hydration speed after the initially delayed hydration speed; and (iii) the introduction of a new material parameter $\tau_{p}$ to calibrate the volume growth on the basis of early age experimental data.

With the transformation function only affecting the early age behavior of the material, with ongoing hydration the modified volume function v(τ(t)) approaches the original volume function v(t). Hence, the calibration scheme proposed in [25] is valid for ages beyond the period of accelerated hydration.

**Figure 1:**
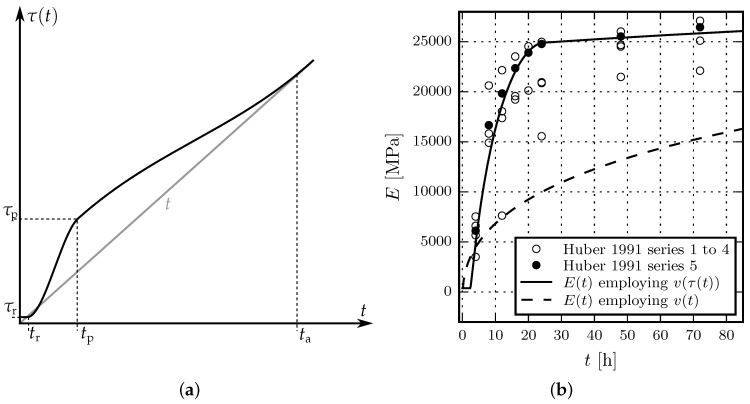
(**a**) Illustration of the time transformation function τ(t) (black curve) versus time *t* (straight gray line); (**b**) Evolution of the Young’s modulus E(t), based on v(t) and v(τ(t)), and comparison with experimental data by Huber [15].

The time transformation function τ(t) is defined as

(19) $$\tau \left(t\right)=\{\begin{array}{cc} \tau_{r} & \mathrm{if}\;t\leq t_{r},\\ \frac{-2\tau_{p}+2\tau_{r}+\Delta t_{p}}{\Delta t_{p}^{3}}{\left(t-t_{r}\right)}^{3}+\frac{3\tau_{p}-3\tau_{r}-\Delta t_{p}}{\Delta t_{p}^{2}}{\left(t-t_{r}\right)}^{2}+\tau_{r} & \mathrm{if}\;t_{r}<t\leq t_{p},\\ \frac{2(\tau_{p}-1)}{\Delta t_{a}^{3}}{\left(t-t_{p}\right)}^{3}-\frac{3(\tau_{p}-1)}{\Delta t_{a}^{2}}{\left(t-t_{p}\right)}^{2}+t-t_{p}+\tau_{p} & \mathrm{if}\;t_{p}<t\leq t_{a},\\ t & \mathrm{otherwise}, \end{array}$$

with $\Delta t_{p}=t_{p}-t_{r}$ and $\Delta t_{a}=t_{a}-t_{p}$. To ensure monotonic growth of τ(t), condition $t_{a}>1.5\;\left(\tau_{p}-1\right)+t_{p}$ must be satisfied. τ(t) and the parameters $\left(t_{r},\tau_{r}\right)$, $\left(t_{p},\tau_{p}\right)$ and $t_{a}$, controlling its behavior, are illustrated in Figure 1a.

The parameters of the transformation function are determined as follows: By assuming $t_{p}=$ 1 d, $\tau_{p}=\tau \left(1\;d\right)$ can be related to the Young’s modulus $E^{\left(1\right)}$ at the age of one day, which can be determined in laboratory tests. $E^{\left(1\right)}$ can be approximated by the inverse compliance, computed for a short time interval Δt according to [25] as

(20) $$E^{\left(1\right)}\approx J{\left(1\;d+\Delta t,1\;d\right)}^{-1}$$

with the compliance function J(t+Δt,t) given according to [25] as

(21) $$J\left(t,t^{\prime }\right)=q_{1}+q_{2}Q\left(t,t^{\prime }\right)+q_{3}\;\ln\left(1+{\left(t-t^{\prime }\right)}^{0.1}\right)+q_{4}\;\ln\left(\frac{t}{t^{\prime }}\right).$$

$J(t,t^{\prime })$ relates a uniaxial constant stress $\bar{\sigma }$, applied at time $t^{\prime }$ to the uniaxial total strain at time *t* as

(22) $$\varepsilon \left(t\right)=\varepsilon^{\mathrm{ve}}\left(t\right)+\varepsilon^{f}\left(t\right)+\varepsilon^{\mathrm{el}}\left(t\right)=J\left(t,t^{\prime }\right)\;\bar{\sigma },$$

assuming linear viscoelastic material behavior and neglecting the shrinkage strain. Equation (22) is based on the integration of (15) for the special case of uniaxial stress, with the initial condition $\varepsilon \left(t^{\prime }\right)=q_{1}\;\bar{\sigma }$.

$Q(t,t^{\prime })$ may be approximated for $t-t^{\prime }\ll t^{\prime }$ according to [20] as

(23) $$Q\left(t,t^{\prime }\right)\approx t^{\prime -0.5}\;\ln\left(1+{\left(t-t^{\prime }\right)}^{0.1}\right).$$

For short durations $\Delta t=t-t^{\prime }$, the term referring to the evolution of the flow strain, i.e., the last term in (21), may be neglected. Substituting (23) into (21) and replacing v(t) by v(τ(t)) in (15), J(1 d+Δt,1 d) can be rewritten as

(24) $$J\left(1\;d+\Delta t,1\;d\right)=q_{1}+q_{2}\;\tau {\left(1\;d\right)}^{-0.5}\;\ln\left(1+\Delta t^{0.1}\right)+q_{3}\;\ln\left(1+\Delta t^{0.1}\right),$$

in which $t^{\prime }$ is replaced by $\tau \left(t^{\prime }\right)=\tau \left(1\;d\right)$. Making use of (20) and (24) and solving for τ(1 d) leads to

(25) $$\tau \left(1\;d\right)=\tau_{p}={\left(\frac{1/E^{\left(1\right)}-q_{1}}{q_{2}\ln\left(1+\Delta t^{0.1}\right)}-\frac{q_{3}}{q_{2}}\right)}^{-2}.$$

Regarding the remaining parameters, experimental data on shotcrete, provided in [15], indicates good agreement by choosing $t_{r}=0.1\;d$ and $\tau_{r}=10^{-2}\;d$. For $t_{a}$, the relation $t_{a}=\max(28,3(\tau \left(1\right)-1)+1)$ ensures a smooth transition to the original function v(t) at a shotcrete age of 28 days or beyond.

Figure 1b shows a comparison of the evolution of the Young’s modulus employing the original volume function and the modified volume function. The compliance parameters, identified from test series 5 in [15], are $q_{1}=16.43$, $q_{2}=206.34$ and $q_{3}=2.61$ (all in 10^−6^ MPa^−1^) and the effective Young’s modulus at the age of 1 day is computed for a time period of $\Delta t=10^{-3}\;d$ as $E^{\left(1\right)}=$ 24,780 MPa. In addition, in Figure 1b, the experimental results for test series 1 to 4 in [15] are shown, which serve for validation of the proposed approach.

### 2.4. Shrinkage

The shrinkage strain is computed by means of the law for concrete, proposed by Bažant and Panula [21], as

(26) $$\varepsilon^{\mathrm{shr}}\left(t\right)=I\;\varepsilon_{\infty }^{\mathrm{shr}}\;k_{h}\;S\left(t-t_{0},\tau_{\mathrm{shr}}\right),$$

in which $\varepsilon_{\infty }^{\mathrm{shr}}$ denotes the ultimate shrinkage strain, $k_{h}$ a humidity-dependent scaling factor, $S(t-t_{0},\tau_{\mathrm{shr}})$ the square-root hyperbolic law, dependent on time *t*, start time of drying $t_{0}$ and shrinkage half time $\tau_{\mathrm{shr}}$, and I, the second order unit tensor. An estimation procedure for determining the material parameters, based on environmental conditions and the concrete mixture, is presented in [21].

## 3. Comparison of the New Shotcrete Model with Other Shotcrete Models

### 3.1. Brief Review of the Shotcrete Models Considered for the Comparison

#### 3.1.1. Viscoplastic Shotcrete Model by Meschke

The total strain *ε* is decomposed into the elastic strain $\varepsilon^{\mathrm{el}}$, the aging induced irrecoverable strain $\varepsilon^{t}$, the shrinkage strain $\varepsilon^{\mathrm{shr}}$, the (visco)plastic strain $\varepsilon^{\mathrm{vp}}$ and the thermally induced strain $\varepsilon^{\theta }$. The evolution of stiffness is modeled by hyperelastic constitutive relations. In this context, the strain is decomposed into a recoverable elastic part $\varepsilon^{\mathrm{el}}$ and an aging induced irrecoverable part $\varepsilon^{t}$. The hyperelastic constitutive relations are specified in total form, relating the stress to the elastic strain by the stiffness tensor determined at the age of 28 days. The evolution of the Young’s modulus is approximated by a modified version of the recommendation of the CEB-FIP model code 1990 [28]. It is based on the material parameters $E^{\left(1\right)}$ and $E^{\left(28\right)}$, denoting the Young’s modulus determined at the age of 1 day and 28 days, respectively, and two parameters $t_{E}$ and $\Delta t_{E}$, controlling the shape of the evolution function. The evolution of the uniaxial compressive strength is described by the ÖVBB recommendation [29] up to the shotcrete age of 24 h and by the relation proposed in [30] afterwards, based on the material parameters $f_{\mathrm{cu}}^{\left(1\right)}$ and $f_{\mathrm{cu}}^{\left(28\right)}$, denoting the uniaxial compressive strength at the age of 1 day and 28 days, respectively. The evolution of the uniaxial tensile strength is also based on the proposal in [30].

Nonlinear mechanical behavior of both hardening and hardened shotcrete is described on the basis of multisurface viscoplasticity theory. A hardening Drucker–Prager model is used for predominant compressive stress states and mixed stress states and a softening Rankine criterion for predominant tensile stress states to model cracking. The plastic strain rate is determined by means of an associated flow rule.

Shrinkage of shotcrete is taken into account on the basis of the semi-empirical model proposed by Bažant and Panula [21], identical to the SCDP model. Creep of shotcrete is modeled by a Duvaut–Lions type viscoplastic formulation. Basically, the viscoplastic strain rate depends on the difference between the stress, computed by assuming elastic material behavior, and the stress, determined by rate-independent plasticity, and a viscosity parameter *η*. Hence, it is assumed that no viscous deformation occurs in the case of elastic material behavior. The model is denoted here as the Meschke model.

#### 3.1.2. Shotcrete Model by Schädlich and Schweiger

The total strain *ε* is decomposed into the elastic strain $\varepsilon^{\mathrm{el}}$ , the shrinkage strain $\varepsilon^{\mathrm{shr}}$, the plastic strain $\varepsilon^{p}$ and the creep strain $\varepsilon^{\mathrm{cr}}$. Aging of shotcrete is considered by evolution equations for stiffness and strength. To this end, the respective equations in the CEB-FIP model code 1990 [28], EN 14487-1 [30,31] are recommended. The evolution laws are based on the values of the Young’s modulus and the uniaxial compressive strength determined at the age of 1 day and 28 days, $E^{\left(1\right)}$ and $f_{\mathrm{cu}}^{\left(1\right)}$, and $E^{\left(28\right)}$ and $f_{\mathrm{cu}}^{\left(28\right)}$, respectively.

Nonlinear mechanical behavior of both hardening and hardened shotcrete is described on the basis of multisurface plasticity theory. A hardening and softening Mohr–Coulomb model is used for predominant compressive stress states and mixed stress states and a softening Rankine criterion for predominant tensile stress states to model cracking. The plastic strain rate is determined by means of a non-associated flow rule.

Shrinkage of shotcrete is taken into account on the basis of the model proposed by the ACI committee 209 [32]. It describes the evolution of the shrinkage strain in terms of the ultimate shrinkage strain $\varepsilon_{\infty }^{\mathrm{shr}}$ and a time-dependent function dependent on a shrinkage half-time parameter $t_{50}^{\mathrm{shr}}$. Creep of shotcrete is modeled on the basis of the theory of viscoelasticity. The evolution of the creep strain is formulated in terms of a creep coefficient $\varphi^{\mathrm{cr}}$, the acting permanent stress and a time-dependent function dependent on a creep half-time parameter $t_{50}^{\mathrm{cr}}$. Nonlinear creep, encountered for higher stress levels, is taken into account. The model is denoted here as the Schädlich model.

#### 3.1.3. Multi-field Shotcrete Model Based on the Hygro–Thermal–Chemo-Mechanical Concrete Model by Gawin et al.

The total strain *ε* is decomposed into the elastic strain $\varepsilon^{\mathrm{el}}$, the creep strain $\varepsilon^{\mathrm{cr}}$, the strain induced by chemical reactions $\varepsilon^{\mathrm{ch}}$, and the thermally induced strain $\varepsilon^{\theta }$. In contrast to the models discussed in the previous subsections, the shrinkage strain is not explicitly contained in this split. It is rather taken into account by the assumption of a multi-phase constitutive effective stress. In order to avoid confusion with the effective stress introduced in the damage-plasticity framework earlier, this constitutive effective stress will be referred to as generalized Bishop stress $\sigma^{\mathrm{Bishop}}$ in the following. Assuming a passive gas phase, it is defined by

(27) $$\sigma^{\mathrm{Bishop}}=\sigma -I\left(a^{\mathrm{Bishop}}\;S_{w}\left(p^{c}\right)+b^{\mathrm{Bishop}}\right)p^{c}.$$

Parameters $a^{\mathrm{Bishop}}$ and $b^{\mathrm{Bishop}}$ are obtained from drying shrinkage tests. Capillary pressure is denoted by $p^{c}$, and $S_{w}$ is the degree of water saturation. The elastic law as well as the creep response are formulated in terms of this generalized Bishop stress. Thus, drying shrinkage manifests in both, contributions to the elastic strain and the creep strain.

Hygral behavior is governed by the van Genuchten law [33], which establishes a relation between the degree of water saturation and capillary pressure. The latter is related to the relative humidity *φ* in the pores based on the Kelvin–Laplace relationship. The two parameters used to describe the sorption characteristics in the van Genuchten law are the air entry value $a^{\mathrm{VanGe}}$ and a dimensionless fitting parameter $b^{\mathrm{VanGe}}$.

The main parameter governing Darcy-type flow through a porous medium is the intrinsic permeability *K*. It is converted to the permeability with respect to the fluid phases using the viscosities of air and water, respectively. For partially saturated conditions, the permeability with respect to a fluid is reduced based on the current degree of saturation. Therefore, relative permeabilities are defined based on the van Genuchten sorption characteristics using Mualem’s approach [34], as usual.

While assuming a constant Poisson’s ratio *ν*, the evolution of other important material properties is described in terms of the degree of hydration Γ [9], such as the uniaxial compressive strength is

(28) $$f_{\mathrm{cu}}\left(\Gamma \right)=f_{\mathrm{cu}}^{\left(\infty \right)}{\left(\frac{\Gamma -\Gamma_{0}}{1-\Gamma_{0}}\right)}^{a_{\mathrm{fc}}},$$

Formulated in terms of the ultimate uniaxial compressive strength $f_{\mathrm{cu}}^{\left(\infty \right)}$, the initial degree of hydration $\Gamma_{0}$, and a power-law exponent $a_{\mathrm{fc}}$. The asymptotic elastic modulus $E_{0}\left(\Gamma \right)$ is defined in the same way, using the ultimate asymptotic elastic modulus $E_{0}^{\left(\infty \right)}$ and a power-law exponent $b_{E}$. Based on a law proposed by Cervera et al. [35] and extended in [9] to take into account the influence of relative humidity on the chemical reaction rate, the rate of the hydration degree is described by an Arrhenius-type law:

(29) $$\begin{array}{c} \dot{\Gamma }=\frac{\left({\hat{A}}_{1}+{\hat{A}}_{2}\;\Gamma \right)\left(1-\Gamma \right)}{1+625{\left(1-\varphi \right)}^{4}}\;\exp\left(-\bar{\eta }\;\Gamma -\frac{5000K}{T}\right). \end{array}$$

It is stated here using a fixed ratio of activation energy and ideal gas constant of 5000 K. The quantities ${\hat{A}}_{1}$, ${\hat{A}}_{2}$, and $\bar{\eta }$ are model parameters and *T* denotes the temperature. Due to the released heat of hydration, the chemical reaction also affects the temperature distribution.

Creep is modeled by the microprestress-solidification theory [36] in a generalized Bishop stress formulation. The short-term, age-dependent, viscoelastic creep response is modeled on the basis of solidification theory. It is based on the same viscoelastic power creep curves with an exponent of 0.1 and parameter $q_{2}$ which are on display in the description of the SCDP model. Since the model proposed in [10] does not have an age-independent contribution to the creep compliance, as it is present in the solidification theory for single-phase materials, the parameter $q_{2}$ in the multi-field model and the SCDP model are not identical in general. To highlight this fact, $q_{2}$ is replaced by $q_{2}^{*}$. Long-term creep is described by means of the microprestress theory. It is characterized by the relaxation of self-equilibriated stresses, i.e., the microprestress, in the cement gel during hydration. If the macroscopic viscosity of the flow creep is assumed to be proportional to the microprestress, the long-term creep is described by three parameters $c^{\mathrm{mps}}$, $c_{0}^{\mathrm{mps}}$, and $c_{1}^{\mathrm{mps}}$. The parameter $c^{\mathrm{mps}}$ governs the viscous flow, while $c_{0}^{\mathrm{mps}}$ controls the evolution of the microprestress in time. They will be fitted from the available shotcrete creep data. A useful interpretation of the ratio $2\;c^{\mathrm{mps}}/c_{0}^{\mathrm{mps}}$, given for instance in [37], is that under standard conditions, this ratio corresponds to the parameter $q_{4}$ present in the solidification theory, which controls the influence of the viscous dashpot part with age-dependent viscosity. Again, $q_{4}$ is replaced by $q_{4}^{*}$ indicating that creep in the multi-field context is based on a different formulation than in the single field models. The influence of changing pore humidity on the evolution of the microprestress is taken into account via $c_{1}^{\mathrm{mps}}$. Since the shotcrete creep data investigated here does not allow to calibrate this value, the value 1.98 MPa determined for concrete in [10,38] is used. A nonlinear dependency of creep on the effective stress at high stress levels is included in the model using the amplification function $F\left(s\right)=\left(1+s^{2}\right)/\left(1-s^{10}\right)$ present for instance in [10,20]. In the present context, due to drying and temperature effects, the stress in the shotcrete specimens is not uniaxial. Therefore, the stress ratio *s* is computed as the ratio between the norm of the (effective) von Mises shear stress and the compressive strength evolving in time.

### 3.2. Evaluation of the Shotcrete Models on the Basis of Experimental Data

In the following, the performance of the SCDP model is evaluated by experimental data from the literature and compared to the performance of the reviewed shotcrete models. Investigated phenomena comprise the evolution of material stiffness and strength, shrinkage and creep. Time-independent nonlinear material behavior for uniaxial and multi-axial stress paths is not addressed here, as it was considered in the respective publications of the models, e.g., based on the biaxial tests by Kupfer et al. [39].

The presented numerical results for the Meschke model, the Schädlich model and the SCDP model are obtained at material point level, prescribing the stress in incremental-iterative simulations based on the respective stress update algorithms. The evolution laws within the framework of plasticity theory are integrated by means of the fully implicit Euler backward scheme within the return mapping algorithm. The evolution laws for creep behavior of the SCDP model and the Gawin model within the framework of viscoelasticity are approximated by Kelvin chains, and are integrated based on the exponential algorithm as proposed in [40]. The respective coefficients of the Kelvin chains are determined from the retardation spectra of the creep compliance functions as presented in [41].

In the multi-field model, mechanical, thermal, and hygral equilibrium is obtained simultaneously in a monolithic way. Since in this approach the variables are not uniformly distributed throughout a specimen, coupled three-dimensional finite element analyses of the considered specimens for determining the displacements, fluid pressures, and temperature have to be performed. For the latter, one-eighth of a cubic specimen is meshed, exploiting symmetry, using 512 elements with quadratic shape functions for displacements and temperature and linear shape functions for capillary pressure. A higher resolution is chosen near the drying surfaces. Thermal and hygral boundary conditions are governed by the ambient temperature $T_{\infty }$ and the ambient relative humidity $\varphi_{\infty }$. The convective heat transfer coefficient is denoted as $\alpha_{c}$ and the convective mass transfer coefficient is named $\beta_{c}$. In the following figures, the results at the center of the specimens are shown.

#### 3.2.1. Comparison of Model Response with Experimental Results by Huber

The experimental data by Huber [15] is chosen for comparing the predicted and measured evolution of the Young’s modulus and the uniaxial compressive strength. The respective experimental data is characterized by a good documentation and a comparatively small scatter of experimental data. In total, five test series, conducted on specimens with dimensions of 0.10 m×0.10 m×0.40 m, are reported. Test series 5 is chosen for the calibration of the shotcrete models, and the numerical results, computed on the basis of those parameters, are compared to the experimental results of the test series 1 to 4. The shotcrete composition, listed in Table 1, is identical for each series.

The measured Young’s modulus at the age of one day in test series 5 is specified in [15] as *E*^(1)^ = 24,780 MPa. Unfortunately, no values for the Young’s modulus at the age of 28 days *E*^(28)^ are reported for any of the test series. For this reason, *E*^(28)^ is estimated as *E*^(28)^ = 30,000 MPa. Parameters $t_{E}$ and $\Delta t_{E}$ of the Meschke model, which govern the early age evolution of stiffness, are identified as $t_{E}$ = 5.65 h and $\Delta t_{E}$ = 4.08 h.

The viscoelastic compliance parameters $q_{2}$ and $q_{3}$ of the SCDP model are computed according to the estimation procedure [25] as $q_{2}=206.34\times 10^{-6}$ and $q_{3}=2.61\times 10^{-6}\;{\mathrm{Mpa}}^{-1}$. To recover $E^{\left(28\right)}=$ 30,000 MPa at the age of 28 days, $q_{1}$ is determined as $q_{1}=16.43\times 10^{-6}$ by computing the effective Young’s modulus based on a duration of $\Delta t=10^{-3}\;d$. Flow compliance parameter $q_{4}$ is not required in the present context.

The simulations using the Gawin model are based on the reported ambient temperature of 23 ${}^{\circ }C$ and ambient relative humidity of 50%. The heat and mass transfer coefficients are $\alpha_{c}=5W/m^{2}\;K$ and $\beta_{c}=0.0002\;m/s$. Thermal conductivity, the densities of solid, air, and water as well as their heat capacities are set to match typical values for ordinary concrete. An intrinsic permeability of 3 × 10^−19^ $m^{2}$ is used, which is considered to be a reasonable guess since it is in the range of values presented in [9] for concrete. The porosity in the fully matured state is assumed to be 12%. The shotcrete specific values calibrated from test series 5 are the parameters governing hydration, the evolution of uniaxial compressive strength and effective Young’s modulus. The values are $\Gamma_{0}=0.02$, ${\hat{A}}_{1}$ = 0.03836 s${}^{-1}$, ${\hat{A}}_{2}=4480.5s^{-1}$, $\bar{\eta }=4.0$, $E_{0}^{\left(\infty \right)}=$ 55,000 MPa, $b_{E}=0.2$, and $q_{2}^{*}=4410^{-6}$. The effective Young’s modulus at the center of the specimen is measured for the same time period Δt as for the SCDP model.

**Figure 2:**
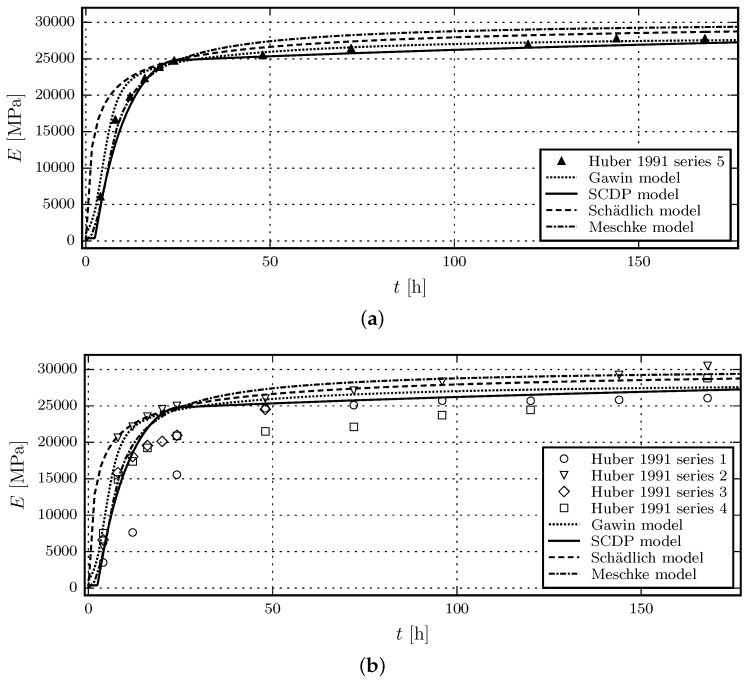
Evolution of the Young’s modulus: (**a**) Results of the calibration of the shotcrete models based on test data of test series 5 by Huber [15]; (**b**) Validation of the shotcrete models by means of the data of test series 1 to 4 in [15].

Figure 2a shows the results of the calibration of the evolution laws for the Young’s modulus based on test series 5 of the experimental data in [15] and Figure 2b contains the validation of the respective evolution laws by means of test series 1 to 4 in [15].

It can be seen that prior to the shotcrete age of 24 h, the evolution of the Young’s modulus, predicted by the Meschke model and the SCDP model, is closer to the experimental data than the one of the Schädlich model. This is mainly due to the considered delayed start of the hydration by both models. Although being based on several assumptions on the hygral and thermal parameters, the predictions using the Gawin model are of a similar quality as the SCDP model and the Meschke model.

The uniaxial compressive strength at the age of one day, $f_{\mathrm{cu}}^{\left(1\right)}$, measured in test series 5, is reported in [15] as 18.2 MPa. Again, as for the Young’s modulus, data for the uniaxial compressive strength at the age of 28 days $f_{\mathrm{cu}}^{\left(28\right)}$ is not available. For this reason, a value of $f_{\mathrm{cu}}^{\left(28\right)}$ 23 MPa is estimated. For the Gawin model, $f_{\mathrm{cu}}^{\left(\infty \right)}=f_{\mathrm{cu}}^{\left(28\right)}$ and $a_{\mathrm{fc}}=1.0$ is assumed.

**Figure 3:**
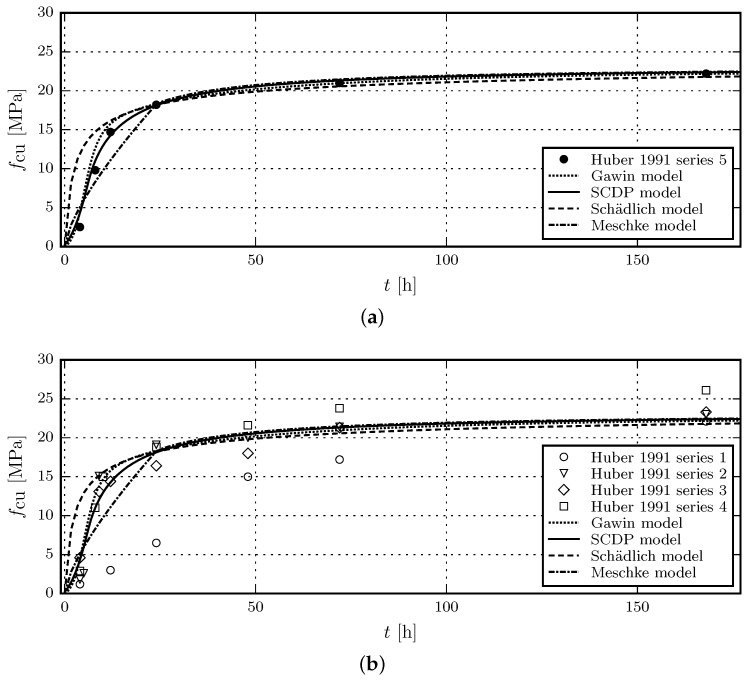
Evolution of the uniaxial compressive strength: (**a**) Results of the calibration of the shotcrete models based on test data of test series 5 by Huber [15]; (**b**) Validation of the shotcrete models by means of the data of test series 1 to 4 in [15].

Figure 3a shows the results of the calibration of the evolution laws for the uniaxial compressive strength based on test series 5 of the experimental data in [15] and Figure 3b contains the validation of the respective evolution laws by means of test series 1 to 4 in [15].

It can be seen that the compressive strength of shotcrete, younger than 24 h, is overestimated by the Schädlich model, while the Meschke model underestimates the strength between 8 and 24 h. The SCDP model is able to represent the delayed start of the evolution of material strength with respect to the casting time, which agrees well with experimental results. Again, the Gawin model and the SCDP model perform equally well. After the age of 24 h, all models predict similar compressive strengths.

#### 3.2.2. Comparison of Model Response with Experimental Results by Müller

The experimental data by Müller [17] is chosen for comparison of the shrinkage and creep behavior of the shotcrete models. Müller presented five series of experimental tests on shotcrete, consisting of four laboratory test series which are compared to one in situ test series. The shotcrete composition is listed in Table 2.

Creep and shrinkage tests were carried out on unsealed specimens from steel molds with dimensions of 0.15 m×0.15 m×0.30 m. Ambient conditions, such as relative humidity or temperature, are not reported.

For the subsequently presented comparison, test series 3 and 4 are chosen because they are characterized by the smallest scatter of data. While test series 3 includes one creep test series, in test series 4, two different loading sequences were applied on two specimens each. The two test schemes are denoted as creep test series 4/1 and creep test series 4/2, which is consistent with the notation used in [17]. The recorded strain from creep tests includes the combined autogenous and drying shrinkage strain as well as the basic and drying creep strain. Hence, shrinkage has to be considered for simulating the creep tests.

The material parameters referring to shrinkage and creep are calibrated on the basis of test series 4 including only creep test series 4/2. Subsequently, the performance of the shotcrete models is evaluated by simulating creep test series 4/1 and 3 employing the identified parameters.

Parameter identification from the shrinkage test on the unsealed specimen in test series 4 by the method of least squares yields the ultimate shrinkage strain $\varepsilon_{\infty }^{\mathrm{shr}}$ = -0.0019 (with $k_{h}$ = 1.0) and the shrinkage half time $\tau_{\mathrm{shr}}$ = 32 d for the Meschke model and the SCDP model. The respective values for the Schädlich model are obtained as $\varepsilon_{\infty }^{\mathrm{shr}}$ = -0.0015 and $t_{50}^{\mathrm{shr}}$ = 8.3 d. Although $\varepsilon_{\infty }^{\mathrm{shr}}$ as well as $\tau_{\mathrm{shr}}$ and $t_{50}^{\mathrm{shr}}$ have the identical physical meaning for all models, different values are estimated. This is the consequence of the different temporal evolution laws of the shrinkage models, which together with the short time span of the experimental shrinkage data, result in the identification of different parameters, depending on the employed shrinkage law.

The environmental conditions, required for the Gawin model, are assumed as $T_{\infty }=23^{\circ }C$ and $\varphi_{\infty }=60\%$. The same heat and mass transfer coefficients, porosity, hygral and thermal parameters as described in the previous subsection for the experiments by Huber are applied. Since drying induces creep in the Gawin model, the drying behavior cannot be calibrated independently from the creep parameters, which are presented for the subsequent numerical simulation of the creep tests.

**Figure 4:**
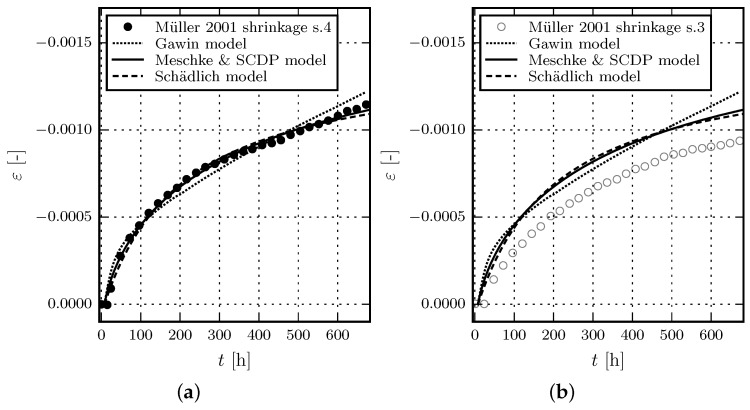
Evolution of the total strain in shrinkage tests: (**a**) Results of the calibration of the shotcrete models based on test data of test series 4 by Müller [17]; (**b**) Validation of the shotcrete models by means of the data of test series 3 in [17].

Figure 4a shows the results of the calibration of the shotcrete models based on test series 4 in [17]. Figure 4b contains the validation of the shotcrete models based on test series 3 in [17].

Creep tests on unsealed specimens were conducted simultaneously with the shrinkage tests. The applied load was increased multiple times during the tests lasting approximately 28 days in total. Periods of constant stress levels lasted only for a few hours or days before the load was increased further. The start time of initial loading and the loading sequence was different for each test series. The individual loading sequences are listed in Table 3. Test series 3 includes identical creep tests on three specimens, here denoted as specimens a, b and c. In test series 4, the two different loading sequences 4/1 and 4/2 were applied to two specimens each. The four specimens of test series 4/1 and 4/2 are denoted as a and b, and c and d, respectively. A third specimen tested in test series 4/1 is not considered here, as the excessive measured total strain indicates a faulty specimen.

The creep parameters of the three models are exclusively identified from test series 4/2, and are subsequently used to simulate test series 3 and 4/1. The Young’s modulus at the age of one day, $E^{\left(1\right)}$, is specified in [17] as $E^{\left(1\right)}$ = 7690 MPa for test series 4. The Young’s modulus at the age of 28 days, $E^{\left(28\right)}$, is computed as the mean value of the two values presented in [17] as $E^{\left(28\right)}$ = 11,580 MPa. The uniaxial compressive strength at the age of one day is reported as $f_{\mathrm{cu}}^{\left(1\right)}$ = 8.72 MPa and the compressive strength at the age of 28 days was measured in tests series 4 on three specimens, from which the mean value is computed as $f_{\mathrm{cu}}^{\left(28\right)}$ = 16.8 MPa.

The ratio of the yield stress over the compressive strength $f_{\mathrm{cy}}/f_{\mathrm{cu}}$, determined from uniaxial compression tests, strongly influences the creep behavior of the Meschke model due to the viscoplastic formulation. Since identification from the creep tests leads to a ratio $f_{\mathrm{cy}}/f_{\mathrm{cu}}$ tending to zero, $f_{\mathrm{cy}}/f_{\mathrm{cu}}$ is estimated as $f_{\mathrm{cy}}/f_{\mathrm{cu}}=0.1$ for all models, which is the lower bound of the range proposed in [23]. The plastic strains at uniaxial compressive peak stress for different shotcrete ages, $\varepsilon_{\mathrm{cpu}}^{p(1)}$, $\varepsilon_{\mathrm{cpu}}^{p(8)}$ and $\varepsilon_{\mathrm{cpu}}^{p(24)}$, are chosen as the default values proposed in [4]: $\varepsilon_{\mathrm{cpu}}^{p(1)}=-30.0\times 10^{-3}$, $\varepsilon_{\mathrm{cpu}}^{p(8)}=-1.5\times 10^{-3}$ and $\varepsilon_{\mathrm{cpu}}^{p(24)}=-0.7\times 10^{-3}$.

Regarding the creep half-time parameter $t_{50}^{\mathrm{cr}}$ of the Schädlich model, identification simultaneously with the creep coefficient $\varphi^{\mathrm{cr}}$ leads to an ill-posed problem due to the short time span of the available experimental data. Hence, $t_{50}^{\mathrm{cr}}$ is chosen as $t_{50}^{\mathrm{cr}}=24\;h$, supported by the evaluation of different values for $t_{50}^{\mathrm{cr}}$ employed for the present parameter identification scheme. For comparison, $t_{50}^{\mathrm{cr}}$ = 36 h is proposed in [42]. The viscoelastic compliance parameters $q_{2}$ and $q_{3}$ of the SCDP model are computed according to the guidelines [25], which results in $q_{2}=269.82\times 10^{-6}\;{\mathrm{Mpa}}^{-1}$ and $q_{3}=3.84\times 10^{-6}\;{\mathrm{Mpa}}^{-1}$, whereas $q_{1}$ follows from the measured value of $E^{\left(28\right)}$ as $q_{1}=59.5\times 10^{-6}\;{\mathrm{Mpa}}^{-1}$, with the effective Young’s modulus computed for a time period of $\Delta t=10^{-2}\;d$. Further creep parameters are identified from both specimens of test series 4/2 considering only the time-dependent strain during the first level of the sustained stress, yielding the viscosity parameter *η* = 16.1 h for the Meschke model, the creep coefficient $\varphi^{\mathrm{cr}}$ = 1.21 for the Schädlich model, and the flow compliance parameter $q_{4}=54.2\times 10^{-6}\;{\mathrm{Mpa}}^{-1}$ for the SCDP model. For the Gawin model, the estimated parameters governing hydration, the evolution of uniaxial compressive strength and effective Young’s modulus are $\Gamma_{0}=0.1$, ${\hat{A}}_{1}=0.01387$ $s^{-1}$, ${\hat{A}}_{2}=1577.7$ $s^{-1}$, $\bar{\eta }=4.0$, $f_{\mathrm{cu}}^{\left(\infty \right)}=17.67$ MPa, $a_{\mathrm{fc}}=1.05$, $E_{0}^{\left(\infty \right)}=$ 28,450 MPa, $b_{E}=0.16$, and $q_{2}^{*}=103\times 10^{-6}\;{\mathrm{Mpa}}^{-1}$. Using this parameter set, the effective Young’s modulus evaluated for a time period of $\Delta t=10^{-2}\;d$ is in the range of 7600 MPa to 7780MPa at the age of 1 day and 11,440 MPa to 11,700 MPa at the age of 28 days. The corresponding values for the uniaxial compressive strength are in the range of 8.57 MPa to 8.9 MPa at the age of 1 day and 16.41 MPa to 17.06MPa at the age of 28 days. Maximum values are always obtained at the center of the sample, while minimum values are located at the corners where drying and cooling slows down the hydration process. Parameters governing the viscous creep are $c^{\mathrm{mps}}=3.1{\mathrm{MPa}}^{-2}\;d^{-1}$ and $c_{0}^{\mathrm{mps}}=0.104{\mathrm{MPa}}^{-1}\;d^{-1}$. The parameters in the effective stress relationship (27) are estimated as $a^{\mathrm{Bishop}}=0.01$ and $b^{\mathrm{Bishop}}=0.16$. They are obtained based on the drying shrinkage test. In the range above 50% water saturation, which is present in the computation, these values model a response which is close to the response of ordinary concrete shown in [10].

**Figure 5:**
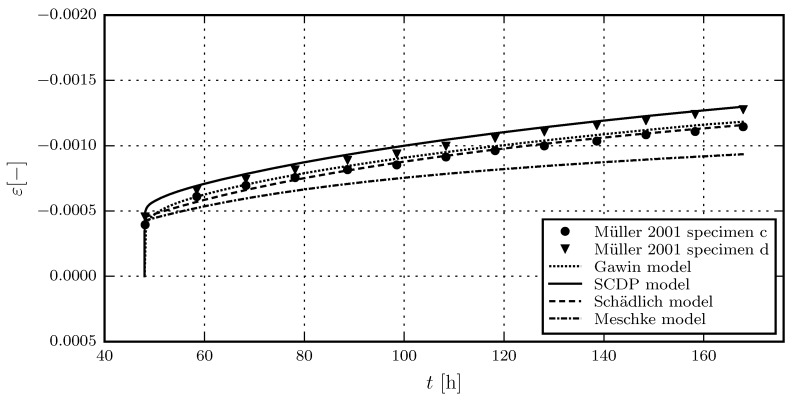
Evolution of the total strain in creep tests on unsealed specimens: Results of the calibration of the shotcrete models based on the test data from creep test series 4/2, load step 1, by Müller [17].

**Figure 6:**
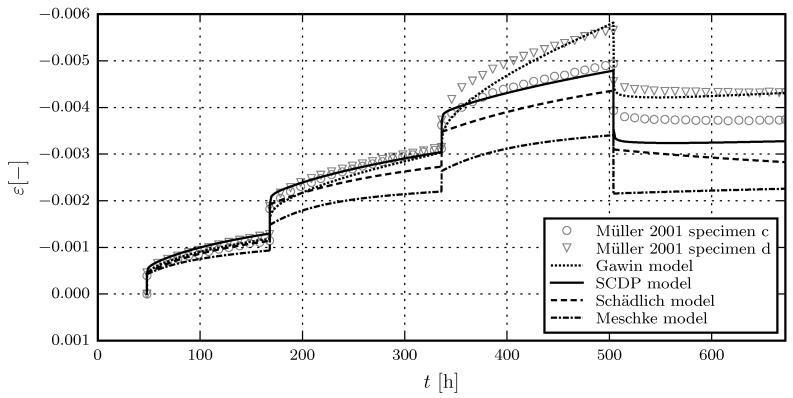
Evolution of the total strain in creep tests on unsealed specimens: Validation of the shotcrete models by means of the test data from creep test series 4/2 by Müller [17].

**Figure 7:**
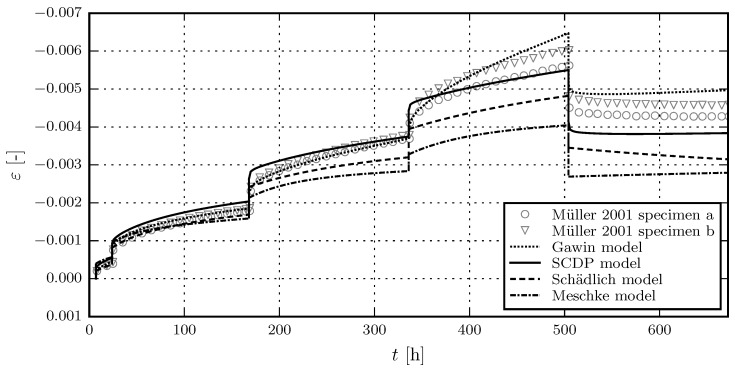
Evolution of the total strain in creep tests on unsealed specimens: Validation of the shotcrete models by means of the test data from creep test series 4/1 by Müller [17].

**Figure 8:**
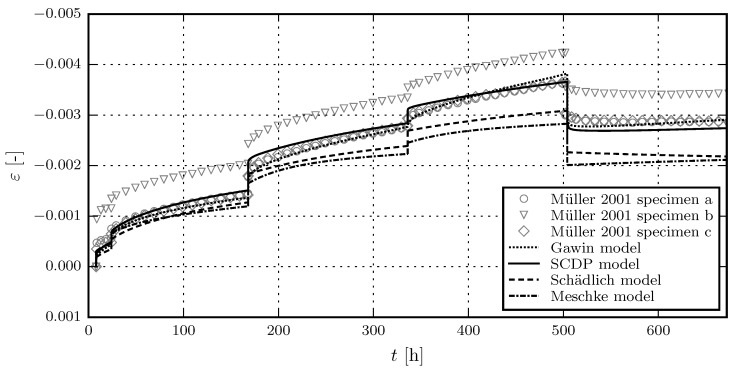
Evolution of the total strain in creep tests on unsealed specimens: Validation of the shotcrete models by means of the test data from creep test series 3 by Müller [17].

Figure 5 shows the results of the calibration of the shotcrete models, based on the experimental data of the first sustained stress level of test series 4/2. Due to the viscoplastic formulation of the Meschke model, the viscosity parameter *η* only controls the rate of the creep strain in the hardening regime, but not its magnitude. This explains the discrepancies between measured and predicted total strain for the Meschke model. The validation of the shotcrete models based on the further load levels of test series 4/2 is shown in Figure 6. Figure 7 and Figure 8 contain the validation of the shotcrete models based on the experimental data of test series 4/1 and 3, respectively.

It can be seen that the numerical results by the SCDP model and the Gawin model agree very well with the experimental data, while the Schädlich model and the Meschke model underestimate the measured total strain. In the Gawin model, the stress-dependent amplification factor for the creep strain at high stress levels, which causes the fast strain increase in the highest load steps of series 4, is very sensitive to the value of uniaxial compressive strength.

## 4. Summary and Outlook

An extended damage plasticity model for shotcrete, able to represent nonlinear time-dependent material behavior, as well as material aging, creep and shrinkage, was proposed. Three well-established constituents serve as the starting point, which are the CDP model by Grassl and Jirásek [18], the solidification theory by Bažant and Prasannan [20] and the shrinkage model by Bažant and Panula [21]. An extension of the CDP model to time-dependent material behavior, as well as a modification of the solidification theory for shotcrete were presented. The material parameters of the new model can be identified from standard experimental tests. Moreover, the identification can be supported by the two parameter estimation procedures presented in [21,25]. The proposed modification of the solidification theory allows for an improved representation of the early age behavior of shotcrete, but maintains the applicability of the calibration scheme proposed in [25] for matured shotcrete.

Based on experimental tests published by Huber [15] and Müller [17], the performance of the model was compared to other models for shotcrete, i.e., the models by Meschke [2], by Schädlich and Schweiger [4] and the multi-field model by Gawin et al. [9,10]. It was shown that the extended damage plasticity model is able to represent the time-dependent material behavior of shotcrete very well.

For future research on the constitutive behavior of shotcrete, it seems to be worthwhile to focus on the nonlinear creep behavior. To identify the nonlinear creep behavior from experimental tests, data of multiple creep tests with different stress levels conducted on specimens of the same age are necessary. However, the experimental data for shotcrete available in the literature is very limited, and many of the available test sets suffer from several shortcomings: (i) Frequently, experimental data on the evolution of the Young’s modulus and the uniaxial compressive strength data are characterized by large scatter. Certainly, this is the consequence of faulty shotcrete specimens caused by the complicated and error-prone in situ casting process; (ii) compressive creep tests frequently consist of several load steps, characterized by only short periods of constant stress levels. This approach seems to be advantageous for the validation of a constitutive model, but it is rather disadvantageous for the identification of creep parameters; (iii) regarding nonlinear creep, to the authors’ best knowledge, no comprehensive experimental data is currently available in the literature; (iv) in general, the measurement periods for the creep tests are too short; it would be beneficial for the calibration process to extend the measurement periods; (v) for many experimental sets, the ambient conditions are not reported, which makes the calibration of a multi-field model virtually impossible. A more accurate calibration of multi-field models would furthermore require shrinkage and creep tests on sealed and unsealed specimens, the determination of the sorption and permeability characteristics as well as convective transfer coefficients, and calorimetric experiments.

Hence, further experimental programs on shotcrete are required for resolving the described deficiencies. 

## Figures and Tables

**Table 1:** Composition of shotcrete for the tests reported by Huber [15].

Property	Quantity	Unit
aggregate content (0/12 mm)	1800	$\mathrm{kg}/m^{3}$
cement content	350	$\mathrm{kg}/m^{3}$
water content	160	$L/m^{3}$
accelerator	57	%

**Table 2:** Composition of shotcrete for the tests presented by Müller [17].

Property	Quantity	Unit
aggregate content (0/8 mm)	1768	$\mathrm{kg}/m^{3}$
cement content *SBM W&P*	340	$\mathrm{kg}/m^{3}$
water content	150	$L/m^{3}$

**Table 3:** Loading sequences for the creep test series 3, 4/1 and 4/2 [17], beginning at casting of the specimens.

Step	Test Series 3		Test Series 4/1		Test Series 4/2	
	Duration	Stress	Duration	Stress	Duration	Stress
**-**	8 h	0 MPa	7 h	0 MPa	48 h	0 MPa
**1**	16 h	-1 MPa	17 h	-1 MPa	120 h	-4 MPa
**2**	144 h	-2.5 MPa	144 h	-4 MPa	168 h	-10 MPa
**3**	168 h	-7.5 MPa	168 h	-10 MPa	168 h	-15 MPa
**4**	168 h	-10 MPa	168 h	-15 MPa	168 h	-0.6 MPa
**5**	168 h	-0.6 MPa	168 h	-0.6 MPa		
