# Correction: An Extended Damage Plasticity Model for Shotcrete: Formulation and Comparison with Other Shotcrete Models

**DOI:** 10.3390/ma11010135

**Published:** 2018-01-15

**Authors:** Matthias Neuner, Peter Gamnitzer, Günter Hofstetter

**Affiliations:** Unit for Strength of Materials and Structural Analysis, Institute of Basic Sciences in Engineering Science, Innsbruck University, Technikerstr. 13, A-6020 Innsbruck, Austria; Peter.Gamnitzer@uibk.ac.at (P.G.); Guenter.Hofstetter@uibk.ac.at (G.H.)

The authors would like to correct following typing errors: For (3) and (4), the correct expressions are given as

(1) $$\begin{array}{c} \begin{array}{cc} f_{p}\left({\bar{\sigma }}_{m},\bar{\rho },\theta ,q_{h}\left(\alpha_{p}\right),t\right)= & \;{\left(\left(1-q_{h}\left(\alpha_{p}\right)\right)\;{\left(\frac{\bar{\rho }}{\sqrt{6}f_{\mathrm{cu}}\left(t\right)}+\frac{{\bar{\sigma }}_{m}}{f_{\mathrm{cu}}(t)}\right)}^{2}+\sqrt{\frac{3}{2}}\frac{\bar{\rho }}{f_{\mathrm{cu}}(t)}\right)}^{2}\\ & +m_{0}\;q_{h}^{2}\left(\alpha_{p}\right)\;\left(\frac{\bar{\rho }}{\sqrt{6}f_{\mathrm{cu}}\left(t\right)}\;r\left(\theta \right)+\frac{{\bar{\sigma }}_{m}}{f_{\mathrm{cu}}(t)}\right)-q_{h}^{2}\left(\alpha_{p}\right), \end{array} \end{array}$$

(2) $$\begin{array}{c} \begin{array}{cc} g_{p}\left({\bar{\sigma }}_{m},\bar{\rho },q_{h}\left(\alpha_{p}\right),t\right)= & \;{\left(\left(1-q_{h}\left(\alpha_{p}\right)\right)\;{\left(\frac{\bar{\rho }}{\sqrt{6}f_{\mathrm{cu}}\left(t\right)}+\frac{{\bar{\sigma }}_{m}}{f_{\mathrm{cu}}(t)}\right)}^{2}+\sqrt{\frac{3}{2}}\frac{\bar{\sigma }}{f_{\mathrm{cu}}(t)}\right)}^{2}\\ & +q_{h}^{2}\left(\alpha_{p}\right)\;\left(\frac{m_{0}\;\bar{\sigma }}{\sqrt{6}f_{\mathrm{cu}}\left(t\right)}+\frac{m_{g}\left({\bar{\sigma }}_{m}\right)}{f_{\mathrm{cu}}(t)}\right), \end{array} \end{array}$$

corresponding to the respective time-independent equations in [1].

For (14), parameter $c_{f}$ is computed as

(3) $$c_{f}=\frac{d\beta_{f}^{\mathrm{II}}}{dt}{|}_{t=t_{f}}-2\;d_{f}\;t_{f}.$$

The authors would like to express their gratitude to Anna-Lena Hammer from Ruhr University Bochum, Germany for finding this error.

The changes do not affect the results. The manuscript will be updated, and the original one will remain available on the article webpage.
